# High-Throughput Metabolic Soft-Spot Identification in Liver Microsomes by LC/UV/MS: Application of a Single Variable Incubation Time Approach

**DOI:** 10.3390/molecules27228058

**Published:** 2022-11-20

**Authors:** Yanlin Zhu, Guiying Chen, Kerong Zhang, Chang Chen, Weiqing Chen, Mingshe Zhu, Hongliang Jiang

**Affiliations:** 1 DMPK Department Shanghai ChemPartner, Shanghai, 201203, China; 2 Tongji School of Pharmacy, Huazhong University of Science and Technology, Wuhan 430074, China; 3 AB Sciex, Beijing 100015, China; 4 MassDefect Technologies, Princeton, NJ 08540, USA

**Keywords:** metabolic soft-spot, LC/UV/MS, metabolite identification, Qtrap mass spectrometry, triple-TOF mass spectrometry

## Abstract

CYP-mediated fast metabolism may lead to poor bioavailability, fast drug clearance and significant drug interaction. Thus, metabolic stability screening in human liver microsomes (HLM) followed by metabolic soft-spot identification (MSSID) is routinely conducted in drug discovery. Liver microsomal incubations of testing compounds with fixed single or multiple incubation time(s) and quantitative and qualitative analysis of metabolites using high-resolution mass spectrometry are routinely employed in MSSID assays. The major objective of this study was to develop and validate a simple, effective, and high-throughput assay for determining metabolic soft-spots of testing compounds in liver microsomes using a single variable incubation time and LC/UV/MS. Model compounds (verapamil, dextromethorphan, buspirone, mirtazapine, saquinavir, midazolam, amodiaquine) were incubated at 3 or 5 µM with HLM for a single variable incubation time between 1 and 60 min based on predetermined metabolic stability data. As a result, disappearances of the parents were around 20–40%, and only one or a few primary metabolites were generated as major metabolite(s) without notable formation of secondary metabolites. The unique metabolite profiles generated from the optimal incubation conditions enabled LC/UV to perform direct quantitative estimation for identifying major metabolites. Consequently, structural characterization by LC/MS focused on one or a few major primary metabolite(s) rather than many metabolites including secondary metabolites. Furthermore, generic data-dependent acquisition methods were utilized to enable Q-TOF and Qtrap to continuously record full MS and MS/MS spectral data of major metabolites for post-acquisition data-mining and interpretation. Results from analyzing metabolic soft-spots of the seven model compounds demonstrated that the novel MSSID assay can substantially simplify metabolic soft-spot identification and is well suited for high-throughput analysis in lead optimization.

## 1. Introduction

Fast metabolism is one of undeniable “Absorption, Distribution, Metabolism, and Excretion (ADME)” prosperities of lead compounds or drug candidates, which could lead to poor bioavailability, fast metabolic clearance, significant drug interaction, and toxic metabolite formation [1,2]. In the past 15 years, the pharmaceutical industry has made great efforts in developing various mass spectrometry-based metabolic stability assays to assess metabolic rates in liver microsomes [3,4], hepatocytes [5,6], and liver S9 [7] in support of lead optimization. Multiple innovative technologies and approaches, such as cassette analysis [8], automation of tandem mass spectrometric method development [9], and RapidFire sample injection [10] were implemented to enhance the throughput of metabolic stability assays. Now, metabolic stability assays in many labs are performed automatically in a high-throughput (HT) fashion using a robotic system for incubation and sample cleanup and fast mass spectrometry analysis lasts from several seconds to a few minutes per sample.

As a part of lead optimization efforts, metabolic soft-spot identification (MSSID) is often conducted for a few metabolized lead compounds that are selected from a large set of compounds predetermined in HT metabolic stability screening. The main goal of MSSID is to determine the metabolic modification site(s) of a testing compound, which are accomplished by identifying one or a few major primary metabolite(s) of the test compound in liver microsomes and hepatocytes [11]. The formation of the metabolite(s) plays a dominant role in metabolic clearance in vitro. The common goals of MSSID assays are the same: to determine the major primary metabolite(s) in vitro and their structures. Results from metabolic stability studies and soft-spot analyses provide critical information for designing more metabolically stable compounds [12,13]. So far, several MSSID assays have been developed for lead optimization in the pharmaceutical industry, which can be categorized into two types of approaches. One approach uses LC/high-resolution mass spectrometry (HRMS) to perform both semi-quantitative analysis and structural characterization of metabolites formed in liver microsomal incubations of test compounds (10–25 µM) for a fixed single or multiple incubation time point(s) [11,14,15,16,17]. Peak areas of metabolites displayed in extracted ion chromatograms are used as a measurement of relative abundances of the detected metabolites. This approach can perform quantitative and qualitative analysis simultaneously; however, quantitative analysis by LC/HRMS often failed to provide a good assessment of major metabolites when ionization efficiencies of different metabolites vary significantly due to matrix effects or significantly different structures. In addition, the incubation experiment with a fixed incubation time, such as 30 min, may generate too many secondary metabolites for very fast metabolized testing compounds, which makes the metabolic soft-spots more complicated. Furthermore, the incubation experiment with multiple time points requires analysis of multiple samples.

To overcome the limitation of the LC/MS-based MSSID assays, an approach using dual-concentration incubation of test compounds in liver microsomes has been developed [18]. A high concentration incubation sample (30 µM, 1 h) is carried first followed by analysis using LC/UV/HRMS to determine the structures and UV/MS correlation factors of individual metabolites. Then, relative concentrations of metabolites in low concentration incubation samples (0.5 µM, multiple time points) are determined using LC/HRMS and the UV/MS correction factors. The dual-concentration approach is capable of generating both metabolic stability and soft-spot data and providing a better quantitative assessment of metabolite formation, but it requires analysis of much more incubation samples than the LC/HRMS-based MSSID assays. Recently, an improved strategy with orthogonal sample-pooling and software-assisted structure elucidation has been implemented in the two-concentration incubation approach, leading to better productivity and turnaround time [19]. 

**Figure 1:**
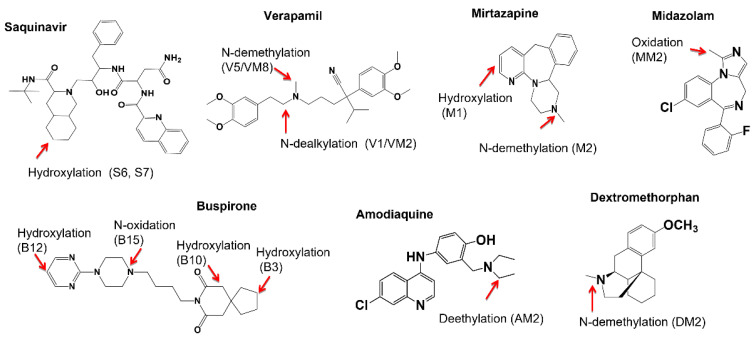
Metabolic soft-spots of seven model compounds, which were determined via HLM incubations (3 or 5 µM) for a compound-specific time followed by analysis using LC/UV/Q-TOF and/or LC/UV/Qtrap.

The major goal of this study was to develop and validate a simple, effective and high-throughput assay for determining metabolic soft-spots of testing compounds in liver microsomes using a single variable incubation time and LC/UV/MS. In the assay performance, test compounds were incubated at contractions of 3 or 5 µM in HLM for a single variable incubation time based on predetermined metabolic stability data. The incubation samples were then analyzed using LC/UVMS and generic data-dependent MS/MS acquisition methods [20,21]. Relative abundances of major primary metabolites formed in the incubations were estimated based on their peak areas in LC/UV profiles. Metabolite detection and structural determination by LC/MS were carried out via data-mining and data interpretation post data acquisitions [22,23,24,25,26,27]. To validate the effectiveness of the single variable incubation time-based assay, metabolic soft spots of seven model compounds (verapamil, dextromethorphan buspirone, mirtazapine, saquinavir, midazolam, and amodiaquine, Figure 1) were determined. Results from this study demonstrated that the novel MSSID assay substantially simplifies experimental procedures in both incubation and sample analysis and significantly improves the result quality of metabolic soft-spot analysis. 

## 2. Results

### 2.1. Workflow of MSSID Assay

**Figure 2:**
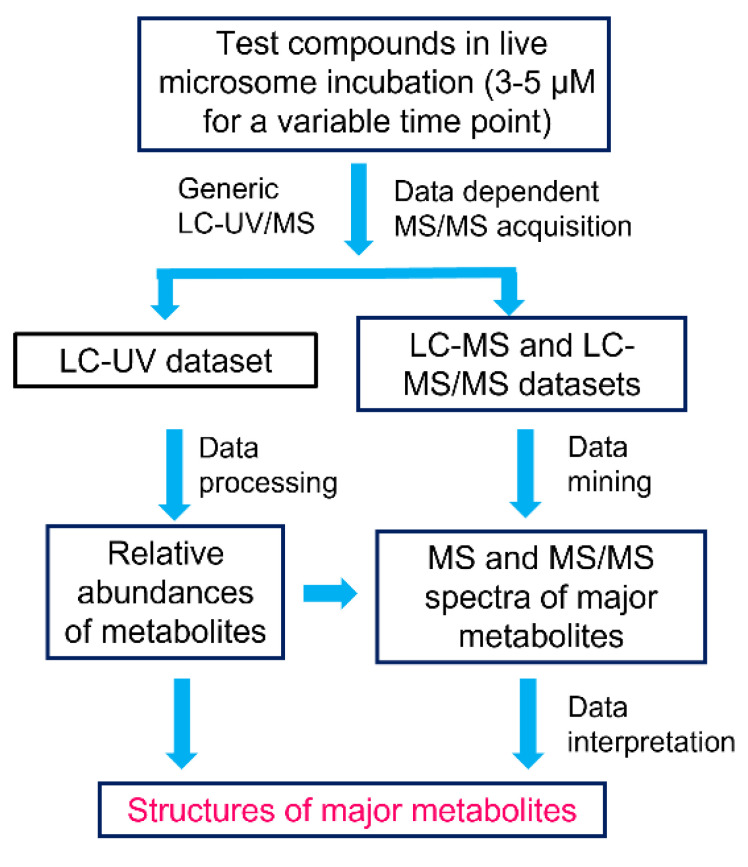
Analytical workflow of metabolic soft spot identification in liver microsomes using LC/UV/MS. Individual test compounds are incubated at 3 or 5 µM for a variable time based on predetermined metabolic stability data. LC/UV/Q-TOF and LC/UC/Qtrap and associated data acquisition and data-mining methods are used for quantitative and qualitative analysis of major metabolites.

The MSSID assay workflow is summarized in Figure 2. Test compounds were incubated at a lower concertation (3 or 5 µM) in liver microsomes for a variable incubation time based on their predetermined metabolic stability data. Basically, a test compound with a shorter metabolic stability half-life value was incubated for a shorter time so that disappearances of individual parent compounds were kept at about 20–40% regardless of their metabolic rates. The parent compound and its metabolites in the incubations were analyzed by LC coupled with a PDA detector and a Q-TOF or Qtrap mass spectrometer. LC/UV profiles, which were generated with selected UV wavelengths or UV ranges based on UV spectra of the individual parent compounds were utilized to assess the relative abundances of metabolites. Presumably, one or a few of the most abundant metabolites formed under the optimal incubation conditions are major metabolite(s), and their formation plays a dominant role in metabolic clearance in liver microsomes. Full-scan MS and MS/MS datasets of the parent compounds and their major metabolites were automatically acquired using a generic acquisition method without optimizing the ionization conditions and acquiring MS/MS spectra of the parent compounds prior to sample analysis. Protonated molecule ions of the major metabolites displayed in LC/UV profiles were directly obtained from the same peaks shown in unprocessed total ion chromatogram (TIC) of full-scan MS datasets or processed LC/MS profiles with EIC, MDF, NLF and/or PIF. Furthermore, MS/MS spectral data of the major metabolites were retrieved from a corresponding MS/MS dataset. Structural elucidation of the major metabolites was accomplished via spectral interpretation.

### 2.2. Metabolic stability of Test Compounds

Half-life values (t1/2) of the seven model compounds in HLM were either determined in this study or obtained from the literature (Table 1). Saquinavir (t1/2 = 2.6 min), amodiaquine (t1/2 = 1.3 min) and midazolam (t1/2 = 3.3 min) were considered as highly metabolized compounds in HLM. Verapamil (t1/2 = 6.9 min) and buspirone (t1/2 = 7.6 min) were compounds with medium clearance. Dextromethorphan (t1/2 = 24 min) and mirtazapine (t1/2 = 165 min) were slowly metabolized in HLM. The metabolic stability data of the model compounds were utilized to set up incubation times (1 to 60 min, Table 1) for the MSSID assay.

### 2.3. Metabolic Soft-Spot Determination Using LC/UV/Q-TOF

**Figure 3:**
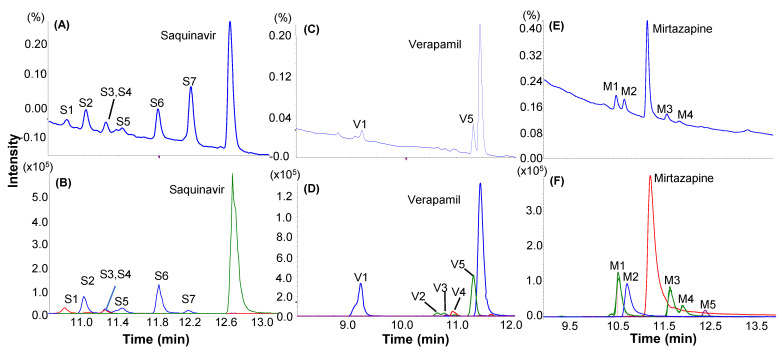
Metabolic soft-spot identification of saquinavir, verapamil, and mirtazapine metabolites in HLM using LC/UV/Q-TOF. (**A**) LC/UV profile of the saquinavir incubation sample (5 µM, 4 min). S6, and S7 had relatively higher abundances and were determined as major metabolites; (**B**) EIC of saquinavir metabolites were found by data-mining; (**C**) LC/UV profile of the verapamil incubation sample (5 µM, 8 min). V1 and V5 were determined as major metabolites; (**D**) EIC of verapamil metabolites were found by data-mining. (**E**) LC/UV profile of the mirtazapine incubation sample (3 µM, 60 min). M1 and M2 were determined as major metabolites; (**F**) EIC of mirtazapine metabolites were found by data-mining.

Metabolic soft-spots of four model compounds, saquinavir, verapamil, buspirone, and mirtazapine, were determined in HLM incubations at 3 or 5 µM for 4, 8, 8, and 60 min, respectively. The HLM incubation samples were then quantitatively and qualitatively analyzed using LC/UV/Q-TOF. As shown in their LC/UV profiles (Figure 3A,C,E and Figure S1A), each of the four parent compounds was the single dominant component, accounting for 58.0–79.3% of the total UV peak areas of all drug-related components (Table 1), indicating that their disappearances were between 21 and 42% by the end of the individual incubation times. The LC/UV profiles revealed one or a few primary metabolite(s) as major metabolite(s) with minimal formation of secondary metabolites. Thus, major metabolites of saquinavir (S6, S7), verapamil (V1, V5), mirtazapine (M1, M3), and buspirone (B3, B10, B12, B15) were assigned, each of which accounted for 6.1–12.1% of the total UV peak areas of all drug-related components (Table 1).

**Figure 4:**
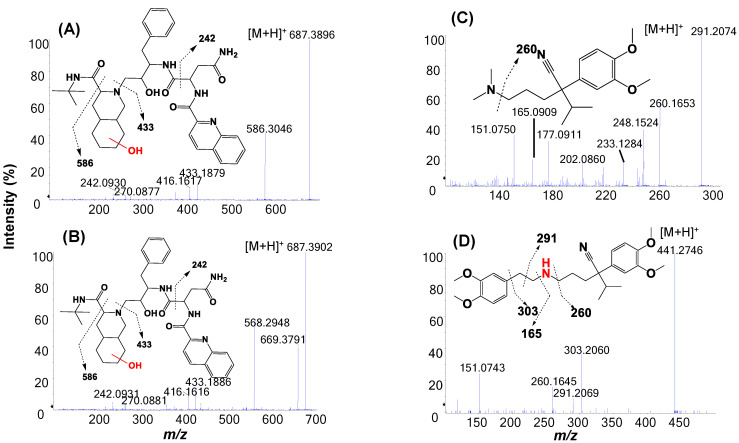
MS/MS spectra of saquinavir and verapamil major metabolites acquired by LC/Q-TOF; (**A**) saquinavir metabolite, S6, (**B**) saquinavir metabolites, S7, (**C**) verapamil metabolite, V1, and (**D**) verapamil metabolite, V5.

LC/MS profiles (extracted ion chromatograms) of the HLM incubations of the four test compounds were displayed in Figure 3B,D,F and Figure S1B. Structural assignments of metabolites detected by LC/HRMS were summarized in Table 1. It should be pointed out that many minor or trace metabolites found by LC/HRMS were not included in Table 1. In general, LC/HRMS analysis was able to detect far more metabolites than those shown in LC/UV profiles. Protonated molecules of major metabolites were readily determined from the peaks displayed in unprocessed LC/MS profiles, which had identical or very similar retention times and peak shapes as compared to major metabolites in LC/UV profiles. Consequently, MS/MS spectra of these metabolites were retrieved from the corresponding MS/MS datasets acquired automatically. Based on full scan MS and MS/MS spectral data, metabolic soft-spots of the four model compounds in HLM were determined (Figure 1, Figure 4 and Figure S2), which are consistent with reported major metabolites of saquinavir, verapamil, buspirone, and mirtazapine in HLM and/or human [18,28,29,30,31]. 

### 2.4. Metabolic Soft Spot Determination by LC/UV/Qtrap

**Figure 5:**
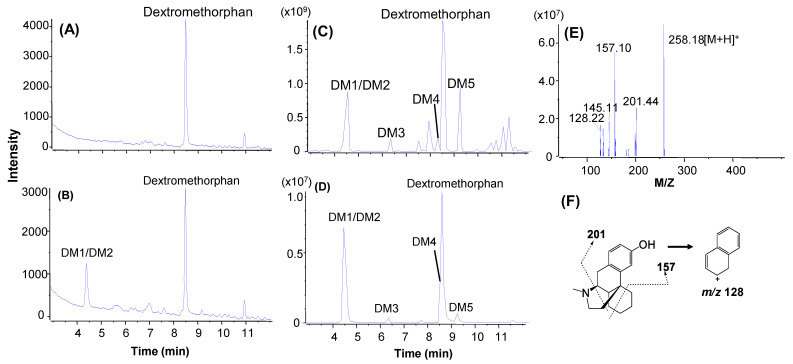
Metabolic soft-spot identification of dextromethorphan in HLM (3 µM, 4 min) by LC/UV/Qtrap. (**A**) LC/UV profile of a dextromethorphan control sample (0 min); (**B**) LC/UV profile of s dextromethorphan incubation sample (30 min); (**C**) LC/MS profile from the MIM-scanning analysis of the dextromethorphan incubation sample (30 min); (**D**) EIC of the dextromethorphan metabolites in the incubation (30 min); (**E**) MS/MS spectrum of DM2; (**F**) structure and fragmentation of DM2.

To evaluate the utility of Qtrap mass spectrometry in the MSSID assay, an LC/UV/Qtrap method was developed and applied to metabolic soft-spot determination of verapamil, dextromethorphan, midazolam, and amodiaquine (Figure 1). The four model compounds were incubated in HLM under same conditions as those described above with a components-specific incubation time from 1 min to 30 min (Table 1). The LC/UV profile of the dextromethorphan incubation sample (3 µM, 30 min) showed two major UV peaks corresponding to the parent drug and coeluted metabolites (DM1 and DM2) accounted for 75.5 and 33.0% of the total drug-related components (Figure 5B and Table 1). An LC/MS profile of the MIM scanning of the sample displayed multiple metabolite peaks (DM1-DM5) (Figure 5C) with a few interference components, from which protonated molecules of these metabolite peaks are directly obtained. In addition, an EIC profile of metabolites detected via data processing was generated (Figure 5D), in which the retention time and shape of the DM1/DM2 peak matched the same peak shown in the LC/UV profile (Figure 5A). Furthermore, MS/MS spectra of the parent drug and its metabolites (Figure 5E,F and Figure S3, Table 1) were readily retrieved from the LC/MS/MS dataset (not shown) that were recorded automatically by MIM-EPI. Based on structures of DM1 (Figure S3) and DM2 (Figure 5F), DM2 was assigned as the single major metabolite of dextromethorphan, which is consistent with the observed major metabolic pathways in humans [32].

**Figure 6:**
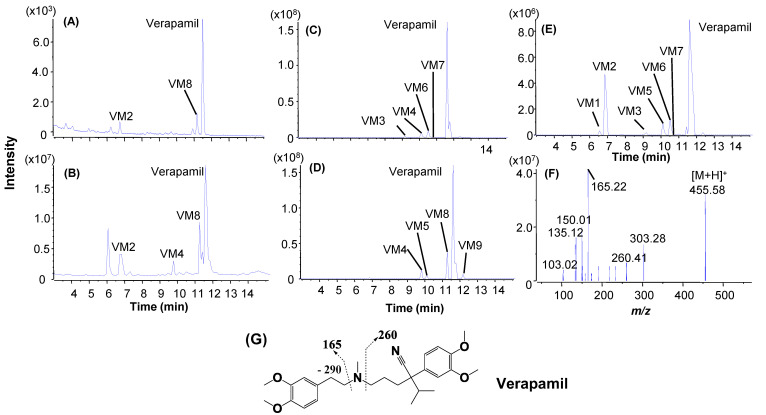
Metabolic soft-spot identification of verapamil (3 µM, 4 min) by LC/UV/Qtrap. (**A**) LC/UV profile; (**B**) LC/MS profile of the MIN scanning; (**C**) LC/MS profile of NLF of 290 Da; (**D**) LC/MS profile of PIF of *m/z* 165; (**E**) LC/MS profile from PIF of *m/z* 260; (**F**) MS/MS spectrum of verapamil; (**G**) major fragmentations of verapamil.

**Figure 7:**
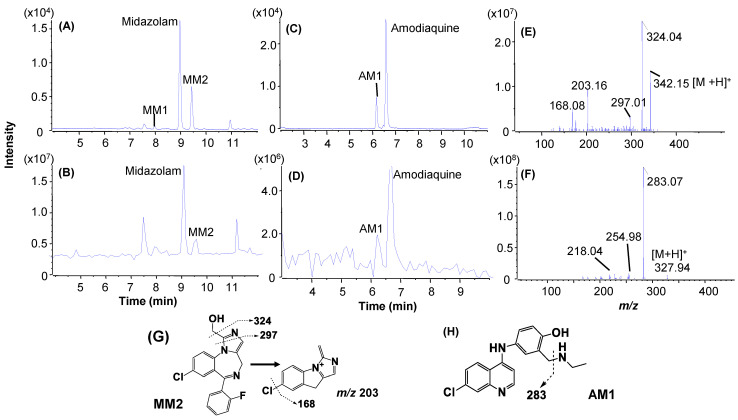
Metabolic soft-spot identification of midazolam in HLM (3 µM, 2 min) and amodiaquine (3 µM, 1 min) by LC/UV/Qtrap. (**A**) LC/UV profile of the midazolam incubation sample; (**B**) LC/MS profile of the MIM scanning of the midazolam incubation sample; (**C**) LC/UV profile of the amodiaquine incubation sample; (**D**) LC/MS profile of MIM scanning of the amodiaquine incubation sample; (**E**) MS2 spectrum of the major metabolite of midazolam (MM2); (**F**) MS2 spectrum of the major metabolite of amodiaquine (AM1); (**G**) structure and fragmentation of MM2; (**H**) structure and fragmentation of AM1.

An LC/UV profile of the verapamil incubation sample (3 µM, 4 min) obtained by LC/UV/Qtrap analysis exhibited two major metabolites, VM2 and VM8 (Figure 6A), along with the parent, consistent with results from the LC/UV analysis of a verapamil incubation sample (5 µM, 8 min, Figure 6). An LC/MS profile of MIM scanning releveled three significant metabolites, VM2, VM4, and VM8, with an interference peak at retention time 6.1 min (Figure 6B). A product ion spectrum of verapamil acquired in the MIM-EPI analysis showed a few characteristic fragmentations (Figure 6F), which were utilized to perform post-acquisition data processing the MS/MS dataset with PIF and NLF (Figure 6G, Table S1). As a result, neutral-loss filtering of 290 Daltons and product ion filtering of *m/z* 260 and 165 led to detection of multiple metabolites in processed LC/MS profiles (Figure 6C–E). Compared to the retention times and peak shapes of the metabolites in the LC/UV to these in the LC/MS profiles, VM2 (N-dealkylated metabolite) and VM8 (N-demethylated metabolite) were assigned as major metabolites of verapamil (Figure 1 and Figure S4), which were consistent with those determined by LC/UV/Q-TOF (Figure 3D). LC/UV profiles of the HLM incubation samples of midazolam (3 µM, 2 min) and amodiaquine (3 µM, 1 min) revelated that both midazolam and amodiaquine formed a single major metabolite, MM2 (Figure 7A) and AM1 (Figure 7C), respectively. Although midazolam and amodiaquine were rapidly metabolized in HLM with very short t1/2 values of 3.3 and 1.3 min, respectively, their disappearances under the tailored HLM incubation conditions were 31 and 23%, respectively (Table 1). The same major metabolites were also displayed in LC/MS profiles from the MIM scanning (Figure 7B,D). Consequentially, full-scan MS and MS/MS spectral data (Figure 7E,F) of the parents and major metabolites were retrieved directly from the LC/MS and LC/MS2 datasets acquired by MIM-EPI, which led to identification of metabolic soft spots of midazolam [33,34] and amodiaquine [35] (Figure 1). 

## 3. Discussion

Usually, a MSSID assay consists of three key components. The first component is the incubation condition, mainly the test compound concentration and incubation time. Single-concentration incubation metabolic soft-port assays in liver microsomes often incubate test compounds at 5–10 µM for either for a single fixed time point between 30 or 60 min, or multiple times points up to 60 min [11]. Dual-concentration MSSID assays perform the first incubation experiment at 30 µM for 30 min, followed by the second incubation experiment at 0.5 µM for seven–eight time points [18]. The second key component is the quantitative method employed to determine major metabolites in the incubation. The single-concertation incubation metabolic soft-spot assays use metabolite peak intensity in extracted ion chromatograms as the measurement of relative abundances of all metabolites detected. On the other hand, the dual-concentration incubation assays obtain UV/MS correction factors of individual metabolites in the higher concentration incubation sample first, then determine relative abundances of the metabolites formed in the low concentration incubation samples using LC/MS and the UV/MS correction factors of individual metabolites. The third component of a MSSID assay is the LC/MS method applied to the detection and structural characterization of metabolites, which involves data acquisition, data mining, and data interpretation [23]. Currently, LC/HRMS instruments and associated data acquisition and data-processing methods, including software-aided spectral interpretation, are mainly used for metabolite profiling and identification in the metabolic soft spot experiments [11,12,13,14,15,18,19,36].

In this study, we developed and evaluated a fit-for-purpose MSSID assay (Figure 2), in which several unique or novel features were implemented to improve the speed and quality of the soft-spot identification experiment. The first unique feature of this MSSID assay was its incubation condition. Test compounds were incubated at a relatively lower concentration (3 or 5 µM) for just a single variable time point that was set up based on metabolic stability data predetermined by a HT metabolic stability assay in liver microsomes. This optimal incubation condition significantly benefited the metabolic soft-spot analysis. First, the formation of metabolites catalyzed by CYP or other metabolizing enzymes in liver microsomes were in linear ranges because the concentrations of the testing compounds were set up at 3–5 µM lower than a majority of Km values of CYP-mediated metabolic reactions. Second, the disappearances of individual parent drugs at the end of the incubations maintained between 20 and 40% regardless of their metabolic rates in liver microsomes (Table 1) so that rates of the metabolism were kept relatively constant during the incubations. Consequently, metabolites with the highest abundances were directly associated with major metabolic clearance pathways in liver microsomes. Third, there was no substantial formation of secondary metabolites in the liver microsomal incubations. Results from analyzing seven model compounds that had diversified structures, major metabolic pathways, and metabolic stability (t1/2 from 1.3 min to 165 min, Table 1) demonstrated that the optimized incubation condition (3–5 µM and a compound-specific incubation time) allowed the maximal formation of one or a few primary metabolite(s) with minimal generation of secondary metabolites (Figure 3A,C,E, Figure 5B, Figure 6A and Figure 7A,C), which fitted the purpose of metabolic soft-spot analysis. 

Many in vitro metabolite identification experiments have been carried out using one or two fixed incubation time(s), such as 30 and 60 min for liver microsome incubations and 2 and 4 h for hepatocyte incubations. These studies are intended to provide complete metabolite profiles of test compounds, including formation of sequential metabolites, in support of metabolism comparison across species and prediction of in vivo metabolism. For example, HLM incubation of nafenodone (10 µM, 60 min) leads to the formation of 26 oxidative metabolites [37]: over 50% of the detected metabolites and two of seven major metabolites are secondary or other sequential metabolites, while the disappearance of the parent was significant (estimated to be over 80%) [38]. However, this type of in vitro experiment with a fixed incubation time may not well suited for metabolic soft-spot analysis of fast metabolized compounds due to the significant formation of secondary metabolites from primary metabolites. Furthermore, it is not necessary to determine the formation time courses for individual metabolites in order to ascertain which are the major metabolites in a MSSID assay. For example, N-dealkylated and N-demethylated verapamil metabolites, V1 and V5 (Figure 3C) were only two major metabolites shown in the LC/UV profile of the HLM incubation of verapamil (5 µM for 8 min). When verapamil is incubated at 0.5 µM with HLM for multiple time points (up to 60 min), V1 and V5 are only two major metabolites at 5 min incubation. Then, a sequential metabolite derived from V1 and V5 becomes the third major metabolite after the incubation times over 10 min [18]. The results from analyzing metabolic soft-sports of the seven model compounds (Figure 1) suggested that this MSSID assay with one optimal incubation time point (Figure 2) can provide equally or more useful information to fit the purpose of metabolic soft-spot identification as compared to that from metabolic soft-spot analysis with a fixed incubation time, such as 30 or 60 min, or multiple incubation times. 

Direct quantitative LC/UV analysis of one single incubation sample to estimate relative abundances of metabolites was the second new feature of this MSSID assay (Figure 2). This approach greatly simplified the analytical perdure for major metabolite identification as compared to the dual-concentration incubation assay, which accomplishes the same goal by performing LC/MS analysis of multiple low-concentration incubation samples and UV/MS correlation factors of individual metabolites predetermined via LC/UV/MS analysis of a high-concentration incubation sample [18]. In general, semi-quantitative analysis of metabolites in the biological matrix without metabolite standards is more accurate using LC/UV than LC/MS since ionization efficiencies of individual metabolites in the LC/MS analysis could be significantly influenced by matrix or their structures.

For example, an LC/UV profile of the HLM incubation of saquinavir displayed two major metabolites S6 and S7 (Figure 3A), consistent of those reported in the literature. In contrast, LC/HRMS profile of the same sample indicated that S7 was a minor metabolite (Figure 3B). Although S6 and S7 were metabolites of mono-oxidation products of saquinavir with similar structures and UV spectra, their fragmentations were significantly different (Figure 4A,B). S7 was readily lost a water (-H_2_O) from a protonated molecule at *m/z* 687.3901 and was a major product ion at *m/z* 586.3048 under CID to generate product ions at *m/z* 669.3791 and 568.2985, respectively. The unique mass spectral characteristic of S7 could be the reason why the amount of S7 was underestimated by LC/MS analysis. 

This metabolic soft-spot assay also implemented unique workflows that allowed for a Triple-TOF and Qtrap mass spectrometer to perform rapid or high throughput structural characterization of one or a few major metabolite(s). The requirements of HT LC/MS analysis in a MSSIS assay include (1) to conduct LC/MS experiment using a generic method without predetermination of MS/MS spectra of the test compounds and optimization of compound-specific analytical conditions using individual test compounds, (2) to record full-scan MS and MS/MS datasets of the parent and its metabolites using a data-dependent or data-independent MS/MS acquisition method in a single injection, and (3) to use software to facilitate detection and spectral interpretation of the major metabolites. LC/HRMS has been exclusively applied to fast metabolic soft-spot identification, while the use of Qtrap in a MSSID assay has not to be reported in the literature. In this study, a set of data acquisition and data-mining tools were assembled to enable LC/Qtrap to perform metabolic soft-spot identification in a high throughput fashion. Full-scan MS and MS/MS spectral data of the parent and its major metabolites in incubation samples were acquired using successive MIM-EPI scanning in a single injection with no requirements for predomination of MS/MS spectral data of the parent compounds or optimized ionization conditions with the parent compounds. Protonated molecules and MS/MS spectra of the parents and their metabolites can be readily retrieved from LC/MS profiles of the MIM scanning and EPI acquisition. Furthermore, the workflow allowed Qtrap to perform the data acquisition of multiple incubation samples continuously. In parallel, data processing and spectral interaction for metabolite identification can be performed. 

The determination of metabolic soft-spots of the four model compounds demonstrated the usefulness of the Qtrap analysis. For example, dextromethorphan had protonated molecule at *m/z* 272.2 so that the MIM-EPI scanning range was set up from *m/z* 50.0 to *m/z* 450.3 that covered all of the potential oxidative metabolites of dextromethorphan. As a result, dextromethorphan metabolites with ion peak intensities above a preset threshold were triggered by the MIM scanning for MS/MS spectral acquisition. Based on the LC/UV (Figure 5B) and LC/MS (Figure 5C,D) profiles, DM2 was immediately assigned as the single major metabolite of dextromethorphan in the HLM incubation. Then, the structure of DM2 was identified as the O-demethylated metabolite based on its MS/MS spectrum and comparison with dextromethorphan (Figure 1). In a similar fashion, one or two major metabolites of verapamil, midazolam, and amodiaquine were rapidly identified and structurally characterized just by comparing major metabolite peaks in LC/UV profiles to those in corresponding LC/MS profiles and interpreting MS/MS spectra of the major metabolites. Qtrap is a hybrid mass spectrometry instrument with both triple quadrupole and ion trap functions and routinely employed for quantitative analysis in support of drug metabolism and pharmacokinetics research, such as analyses of samples from in vitro metabolic stability [39] and animal pharmacokinetics [40] studies. The development of Qtrap-based metabolic soft-spot assay in this study and other metabolite identification methods [20,24,26,41,42,43,44] would allow Qtrap to be used as a single LC/MS platform for both quantitative and qualitative analysis, which is beneficial to DMPK or bioanalysis labs in terms of saving time and resources. 

## 4. Materials and Methods

### 4.1. Chemicals and Reagents

Buspirone, mirtazapine, saquinavir, and verapamil were purchased from Sigma (St. Louis, MO, USA). Midazolam, 1’-hydroxyl-midazolam, nomifensine, and dextromethorphan were purchased from National Institutes for Food and Drug Control (Beijing, China). Amodiaquine and N-desethyl amodiaquine were purchased from Toronto Research Chemical Inc. (North York, ON, Canada). β-nicotinamide adenine dinucleotide 2′-phosphate reduced tetrasodium salt (NADPH) was purchased from Beijing Dingguochangsheng Biotechnology Co., LTD (Beijing, China). All solvents (acetonitrile, methanol, and water) were of high-performance liquid chromatography grade and purchased from Fisher Scientific (Pittsburgh, PA, USA). Pooled HLM was obtained from BD Biosciences (Woburn, MA, USA).

### 4.2. Determination of Metabolic Stability in HLM

Midazolam, dextromethorphan, verapamil and amodiaquine (0.5 µM) were incubated separately with HLM (0.5 mg/mL) in phosphate buffer (100 mM; pH 7.4) at 37 °C. NADPH (1 mM) was added to initiate metabolic reactions after a 5.0 min pre-incubation. At specific reaction time points (0, 2, 5, 10, 20, and 30 min for midazolam, dextromethorphan, verapamil, and 0, 1, 2, 3, 5, and 10 min for amodiaquine), 0.1 mL aliquots of the incubation samples were mixed with a 2-fold volume of ice-cold acetonitrile containing the internal standard (100 ng/mL nomifensine). The samples were then centrifuged at 13,300× *g* at 4 °C for 10 min, and 2 µL supernatants were analyzed by LC/Triple TOF for relative quantitation of remaining parent compounds. In addition, the T1/2 value of saquinavir (1 µM) was determined in HLM under similar incubation conditions using a linear ion trap mass spectrometer.

The LC/MS system consisted of the LC instrument (Shimadzu HPLC system with a UV detector SPD20A, Kyoto, Japan) and a Qtrap mass spectrometer (API 4500 Qtrap mass spectrometer (Sciex, Framingham, MA, USA). Electrospray ionization (ESI) was used to produce ions for mass spectrometry detection. An AQUASIL C18 column (50 mm × 2.1 mm, 5 µm, Thermo Electron, Waltham, MA, USA) was used to separate analytes under a gradient of 0.1% formic acid and 5.00 mM ammonium formate in water (mobile phase A) versus acetonitrile (mobile phase B) at a flow of 0.8 mL/min. The HPLC gradient was set as follows: 0–0.2 min, 10% B; 0.2–1.0 min, 10–95% B; 1.0–1.8 min, 95% B; 1.8–1.9 min, 95–10% B; 1.9–2.5 min, 10% B. MRM was used as data acquisition method and the details were shown in Table S2. Relative quantitation of parent drugs was achieved by peak integration of the extract ion chromatograms (EICs).

### 4.3. Determination of Metabolic Soft Spots in HLM Using LC/UV/HRMS

Buspirone (3 µM), mirtazapine (3 µM), saquinavir (5 µM), and verapamil (5 µM) were incubated in HLM by the following the same procedure as the metabolic stability assay. Incubation times were 4 min for buspirone, 8 min for saquinavir and verapamil, and 60 min for mirtazapine, which were set up based on predetermined metabolic stability T1/2 values in HLM (Table 1). Supernatants of incubation samples were dried under nitrogen and then reconstituted in 100 μL of 5% acetonitrile in water prior to analysis using LC/UV/HRMS. 

An LC/MS system consisted of an LC instrument (Shimadzu HPLC with a photodiode array detector (PDA), Shimadzu, Kyoto, Japan) and TripleTOF (AB 4600, Sciex, Framingham, MA, USA) was employed. LC separation was carried out using a Phenomenex Kinetex-C18 (2.1 × 150 mm, 2.6 μm, CA) and a mobile phase comprised of 0.1% formic acid (*v*/*v*) in water (solvent A) and 0.1% formic acid in acetonitrile (*v*/*v*) (solvent B). The total running time was 5 min, and the flow rate was 0.4 mL/min. A linear gradient was set as below: 0–0.6 min, 10% B; 0.6- 1.3 min, 10% to 50% B; 1.4–1.7 min, 50% to 90% B; 1.7–2.3 min, 90% B; 2.3–5 min, 10% B. The PDA detector was set to collect UV data across the wavelength range of 190 to 350 nm. The mass spectrometer was operating in the positive electrospray ionization mode in the *m/z* range of 100–1000. Dynamic background subtraction (DBS) was employed to record full-scan MS and MS/MS data simultaneously. Two approaches were used for finding and confirming protonated molecules of major metabolites. One approach was to directly get the information by reviewing full-scan MS spectra of peaks in unprocessed LC/MS dataset, which had very similar LC retention times and peak shapes to those of major metabolites shown in corresponding LC/UV profiles. Another approach was to process full scan MS datasets using MDF and EIC tools [22,25] on MetabolitePolit software (Sciex, Framingham, MA, USA) and then to compare retention times and peak shapes of major metabolite(s) shown in LC/UV profile to metabolite peaks displayed in processed LC/MS profiles. The structural characterization of metabolites was carried based on interpretation of MS/MS spectra that were acquired in the same injection (Figure 1).

### 4.4. Determination of Metabolic Soft-Spots in HLM Using LC/UV/Qtrap

Midazolam, dextromethorphan, verapamil, and amodiaquine were incubated at 3 μM with HLM under the same conditions described above. A specific incubation time for the individual test compounds was set up for 1–20 min based on their metabolic stability T1/2 values (Table 1). An LC/UV/Qtrap system with an Ultimate XB-C18 column (100 mm × 2.1 mm, 1.8 µm, Welch, China) was employed for quantitative and qualitative analyses of metabolites of the test compounds in HLM incubations. The mobile phases were the same as the metabolic stability assay, and the flow rate was 0.3 mL/min. The gradient was set as follows: 0–2 min, 15% B; 2–12 min, 15–45% B; 12–13 min, 45–90% B; 13–16 min, 90% B; 16–16.1 min, 90–15% B; 16–20 min, 15% B. The on-line UV detector was set up at a wavelength range of 200–450 nm. The mass spectrometer was operated in positive ion mode using a successive multiple ion monitoring-triggered enhanced product ion scan (MIM-EPI) mode [26,27]. The successive MIM scanning range was *m/z* 50.0–450.3 for midazolam, dextromethorphan, and amodiaquine and *m/z* 150.1–550.3 for verapamil. All the ion source parameters were set as the following values: CUR: 30 psi; IS: 5500 V; TEM: 500 °C; GS1 and GS2: 50 psi; DP: 70 V. A criterion for the data-dependent EPI was set for the three most intense ions in each dynamic background subtraction (DBS) survey scan spectrum with an intensity threshold of 500 cps. The CE was set to 5 eV for the survey scan and 40 eV with CE spread (CES) of 15 eV for the dependent EPI scan. The scan speed for ER and EPI were 250 amu/s and 20,000 amu/s, respectively. LC/UV profiles of individual incubation were generated to reveal major metabolites using wavelengths on the λmax of parent compounds (Table S1). Protonated molecules of major metabolites were determined by matching major metabolite peak(s) in LC/UV profiles to similar ion peaks shown in LC/MS profiles from the MIM scanning or data processing. EIC, PIF, and NLF tools on PeakView SoftwareTM 1.2 (AB SCIEX, Foster City, CA, USA) were used to process LC/MS and LC/MS2 datasets. MS/MS spectral data of major metabolites were retrieved from corresponding LC/MS2 data based on their protonated molecules. The structural characterization of major metabolites was carried based on interpretation of their MS/MS spectra and biotransformation knowledge.

## 5. Conclusions

In this study, a novel soft-spot identification assay featured with a compound-specific incubation time and direct LC/UV/MS analysis was developed. In the analysis using the assay, test compounds were incubated at low contraction of 3 or 5 µM in HLM for a single variable incubation time based on predetermined metabolic stability data. The incubation samples were then analyzed by LC/UV coupled with Q-TOF or Qtrap mass spectrometry. Relative abundances of one or a few major primary metabolites formed in the incubations were estimated using on the basis of their peak areas in LC/UV profiles. Metabolite detection and structural characterized were carried out using on-line Q-TOF or Qtrap and generic data-dependent MS/MS acquisition methods. Metabolite detection and spectral interpretation can be performed post data acquisition without reinjections of the same incubation samples. 

Results from analyzing seven model compounds using the assay showed that the optimal incubation condition generated unique metabolite profiles, in which patents accounted for 60–80% of the total UV peaks of drug-related components, one or a few primary metabolites associated with metabolic soft-spots were the most abundant metabolites, and there were no significant secondary metabolites regardless of their metabolism rates. The metabolite profiles greatly simplified the metabolic soft-spot analytical procedure and allowed LC/UV to directly perform semi-quantitation for identifying major metabolites without using UV/MS correction factors. Furthermore, LC/Q-TOF and LC/Qtrap were enabled to perform automated acquisition of full-scan MS and MS/MS datasets of the parents and their metabolites in a single LC/UV/MS injection, from which molecular ions and MS/MS spectral data of the parent compounds and major metabolite were readily retrieved from unprocessed or processed LC/MS profiles. Compared to commonly used metabolic soft-spot identification assays, the new MSSID assay required minimal sample analysis (usually a control sample from zero time point and a test sample from one tailored incubation time), and minimal metabolites for structural characterization (usually one or two major metabolite(s)). Since major metabolites generated under the incubation conditions had relatively higher concentrations and MS/MS spectral data of the parent compounds can be acquired together with metabolites in the same LC/UV/MS analysis, the predetermination of optimized ionization conditions or MS/MS spectra of parent compounds were not needed. Therefore, LC/UV/MS analysis of a large number of incubation of samples can be continuously performed without second injections or interruptions for setting up different acquisition methods or data processing. Together with software-aided spectral interpretation, this metabolic soft-spot assay is applicable to high-throughput identification of metabolic soft-spots of multiple compounds after prescreen with metabolic stability experiments in lead optimization.

## Figures and Tables

**Table 1:** Quantitative determination and structural characterization of metabolites of testing compound in HLM incubation by LC/UV/Triple TOF or LC/UV/Qtrap. Major metabolites were formed via metabolic reactions on metabolic soft spots.

Compound(T1/2) ^a^	Incubation Conditions	The Parent Drugs (Relative Abundances)	Major Metabolites and (Relative Abundances ^b^)	Minor or Trace Metabolites ^c^
**Analyzed using LC/UV/Triple TOF**				
Saquinavir ^a^ (2.6 min)	5 μM, 4 min	Saquinavir (58%)	Mono-oxidation: S6 (11.6%), S7 (12.8%).	Di-oxidation: S1, S3, S8, S9; Mono-oxidation: S4, S5, S10.
Verapamil ^a^ (6.9 min)	5 μM, 8 min	Verapamil (79.3%)	N-dealkylation: V1 (6.1%); N-demethylation: V5 (14.7%);	O-demethylation: V2, V3; Mono-oxidation: V4.
Mitazapine ^d^ (165 min)	3 μM, 60 min	Mitazapine (74.2%)	Momo-oxidation: M1 (10.7%); N-demethylation: M2 (8.1%); N-oxidation: M3 (5.0%).	Mono-oxidation: M4; Ketone formation: M5.
Buspirone ^d^ (7.6 min)	3 μM, 8 min	Buspirone (59.1%)	Mono-oxidation: B3 (12.1%), B10 (16.5%), B12 (5.2%), B15 (7.1%).	N-dealkylation: B1 ^e^. Mono oxidation: B4, B7; Di-oxidation: B2, B5, B6, B8, B9, B11, B14; Desaturation: B13.
**Analyzed using LC/UV/Qtrap**				
Midazolam ^a^ (3.3 min)	3 μM, 2 min	Midazolam (69.0%)	Mono-oxidation: MM2 (29.0%)	Hydroxylation: MM1
Dextromethorphan ^a^ (24.0 min)	3 μM, 30 min	Dextromethorphan (75.5%)	Demethylation: DM1/DM2 (33.0%).	N-demethylation: DM4Hydroxylation: DM3, DM5
Amodiaquine (1.3 min) ^a^	3 μM, 1 min	Amodiaquine (77.0%)	Deethylation: AM1 (26.5%)	Not detected
Verapamila (6.9 min)	3 μM, 4 min	Verapamil (72.1%)	N-dealkylation: VM2 (8.9%);N-demethylation: VM8 (19.0%)	N-dealkylation+ N-demethylation + hydroxylation: VM1; N-dealkylation: VM3. O-demethylation + hydroxylation: VM4, VM9; N-demethylation + O-demethylation: VM5, VM7; O-demethylation: VM6, VM11; Hydroxylation: VM10, VM13; N-demethylation: VM12

^a^ T1/2 value was determined in the current study. ^b^ Relative abundances of major metabolites and the parent drugs were expressed as % of the total of UV peak areas of drug and its metabolites displayed in LC/UV profiles. ^c^ Minor or trace metabolites. Minor metabolites had relatively small UV peaks and trace metabolites that were detected by LC/MS but not displayed in LC/UV profiles. ^d^ The T1/2 value is from the reference [18], in which a test compound (0.5 µM) was incubated with HLM (0.5 mg/mL) for 60 min followed by LC/MS analysis of the parent drug. ^e^ B1 should be one of major metabolite of buspirone formed in HLM (Ref Zhu et al. DMD). However, its UV peak was not clearly displayed in this study due to overlapping with endogenous components.

## Data Availability

Not applicable.

## Supplementary Materials

The following supporting information can be downloaded at: https://www.mdpi.com/article/10.3390/molecules27228058/s1, Figure S1: quantitative and qualitative analysis of buspirone metabolites formed in HLM incubation (3 µM, 8 min) by LC/Q-TOF. (A) LC/UV profile of the buspirone incubation. B3, B10, B12, and B15 were determined as major metabolites of buspirone. (B) EIC of buspirone metabolites; (C) EIC of buspirone metabolite B1.; Figure S2: MS/MS spectra and fragmentations of mirtazapine metabolites acquired by LC/Q-TOF. (A) M1, (B) M2 and (C) M3; Figure S3: MS/MS spectra and fragmentations of dextromethorphan (A) and its metabolites, DM1 (B), and DM4 (C) acquired by LC/UV/Qtrap; Figure S4: MS/MS spectra and fragmentations of verapamil major metabolites VM2 and VM8 acquired by LC/Qtrap. (A) VM2, and (B) VM8; Table S1: summary of metabolites of verapamil detected by Qtrap and data mining of NLF and PIF; Table S2: the MRM transitions, mass parameters and absorption wavelength (λmax) of the model compounds.

## Sample Availability

Samples of the compounds are not available from the authors.
