# Supplementary material for: High-Throughput Metabolic Soft-Spot Identification in Liver Microsomes by LC/UV/MS: Application of a Single Variable Incubation Time Approach
Source: Molecules. 2022 Nov 20;27(22):8058. doi: 10.3390/molecules27228058 (PMC9693510; doi:10.3390/molecules27228058)
Supplement: Supplementary file 1 [file molecules-27-08058-s001.zip › molecules-1962071-supplementary.pdf]

Support Information

Table of Contents

Support Figures **2**

Support Table **6**

Support Figures

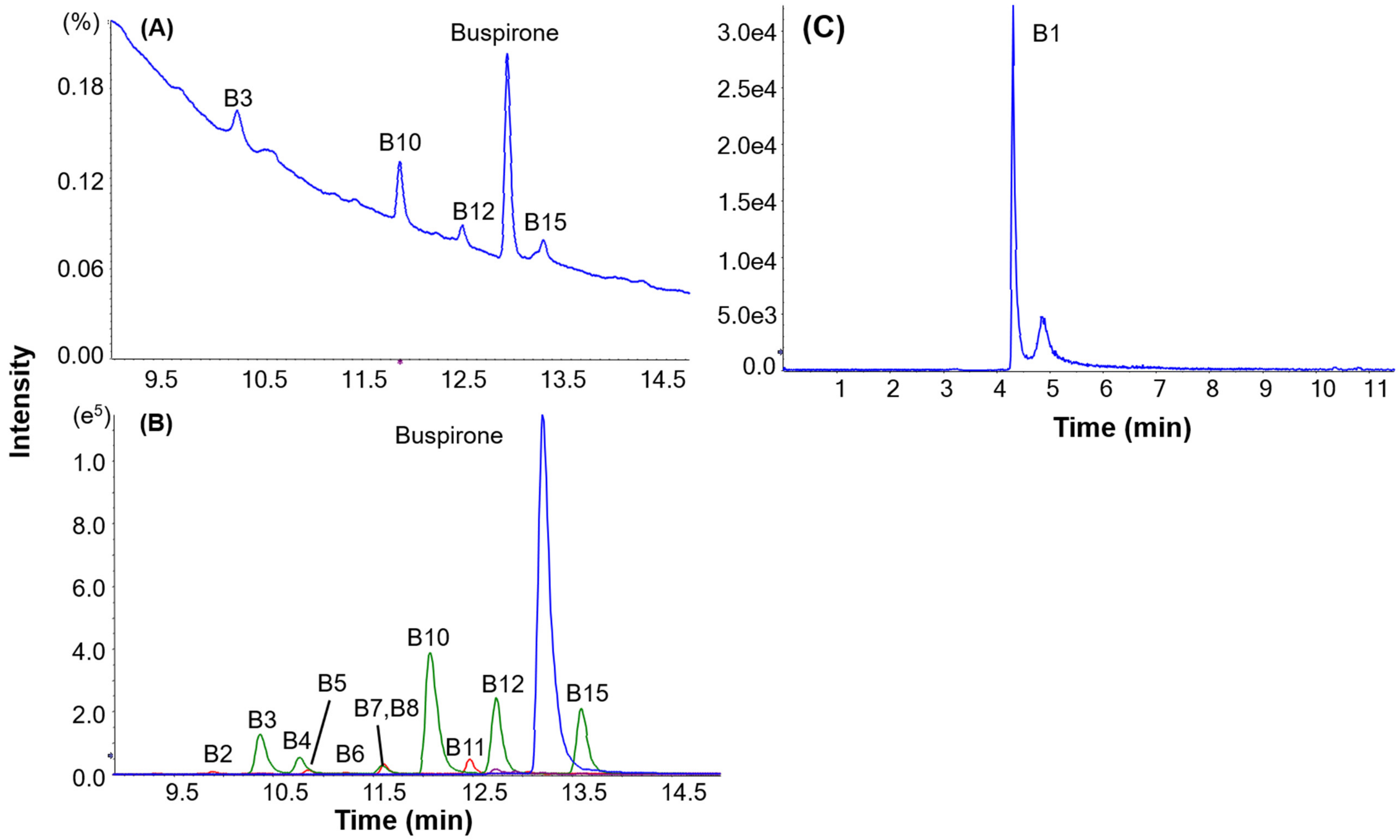

Figure S1. Quantitative and qualitative analysis of buspirone metabolites formed in HLM incubation (3 μM, 8 min) by LC/Q-TOF. (A) LC/UV profile of the buspirone incubation. B3, B10, B12, and B15 were determined as major metabolites of buspirone. (B) EIC of buspirone metabolites; (C) EIC of buspirone metabolite B1.

Fig. S2.

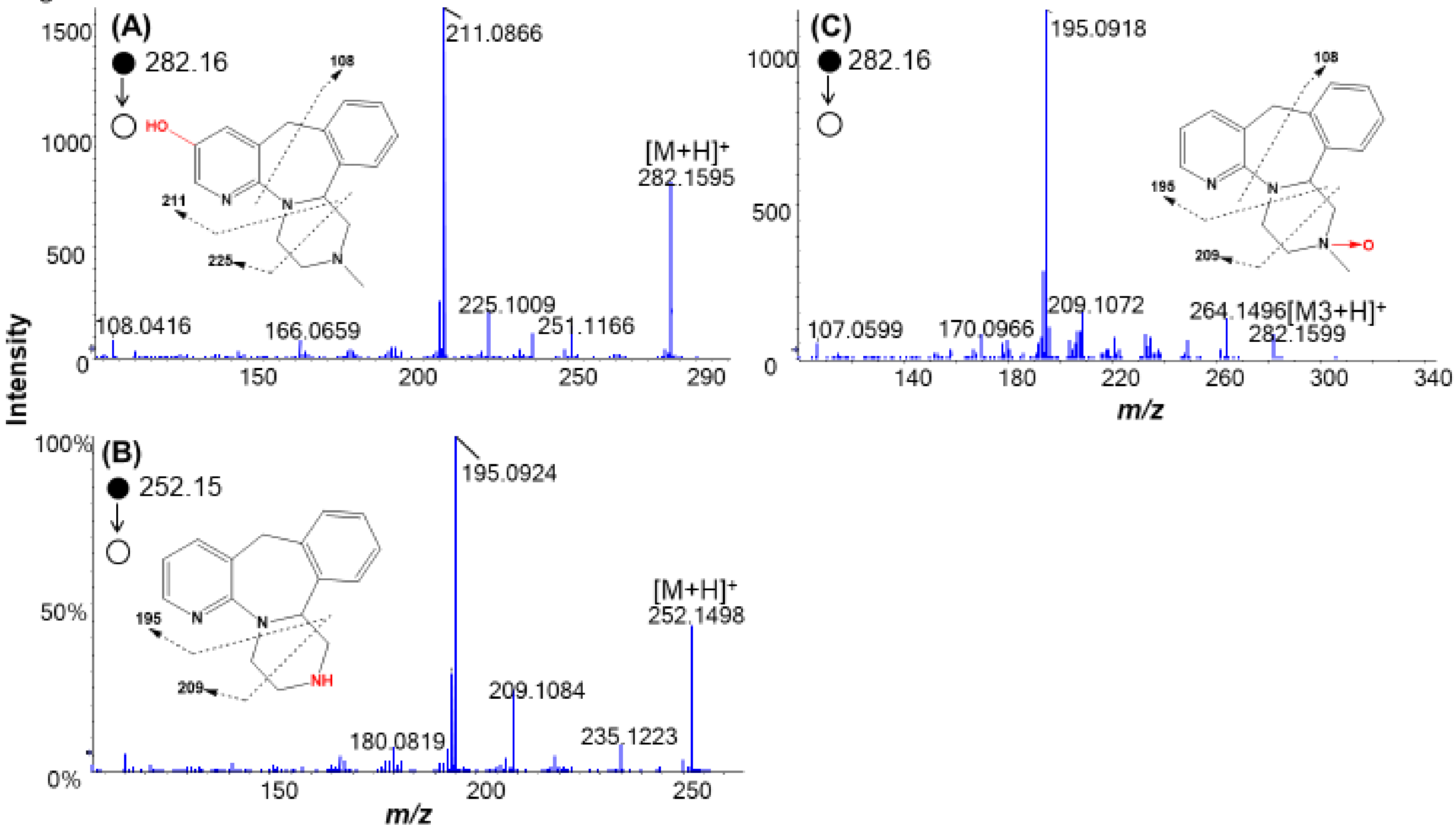

Figure S2. MS/MS spectra and fragmentations of mirtazapine metabolites acquired by LC/Q-TOF. (A) M1, (B) M2 and (C) M3.

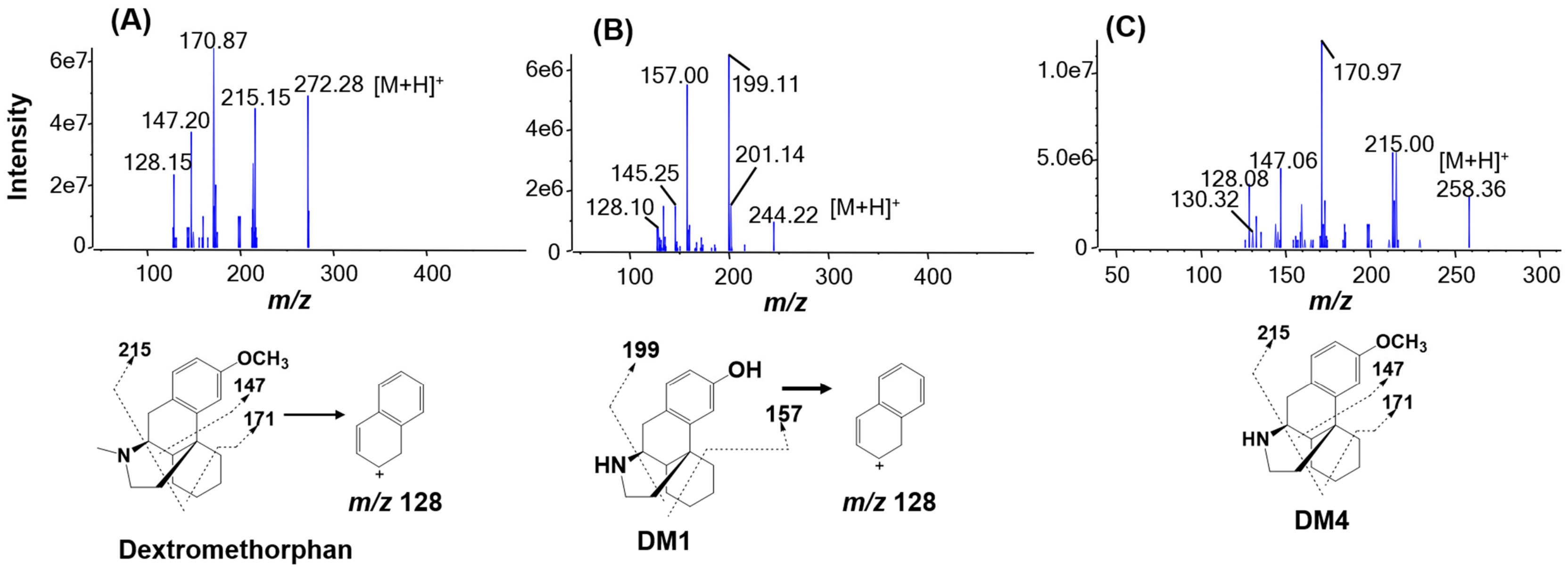

Figure S3. MS/MS spectra and fragmentations of dextromethorphan (A) and its metabolites, DM1 (B), and DM4 (C) acquired by LC/UV/Qtrap.

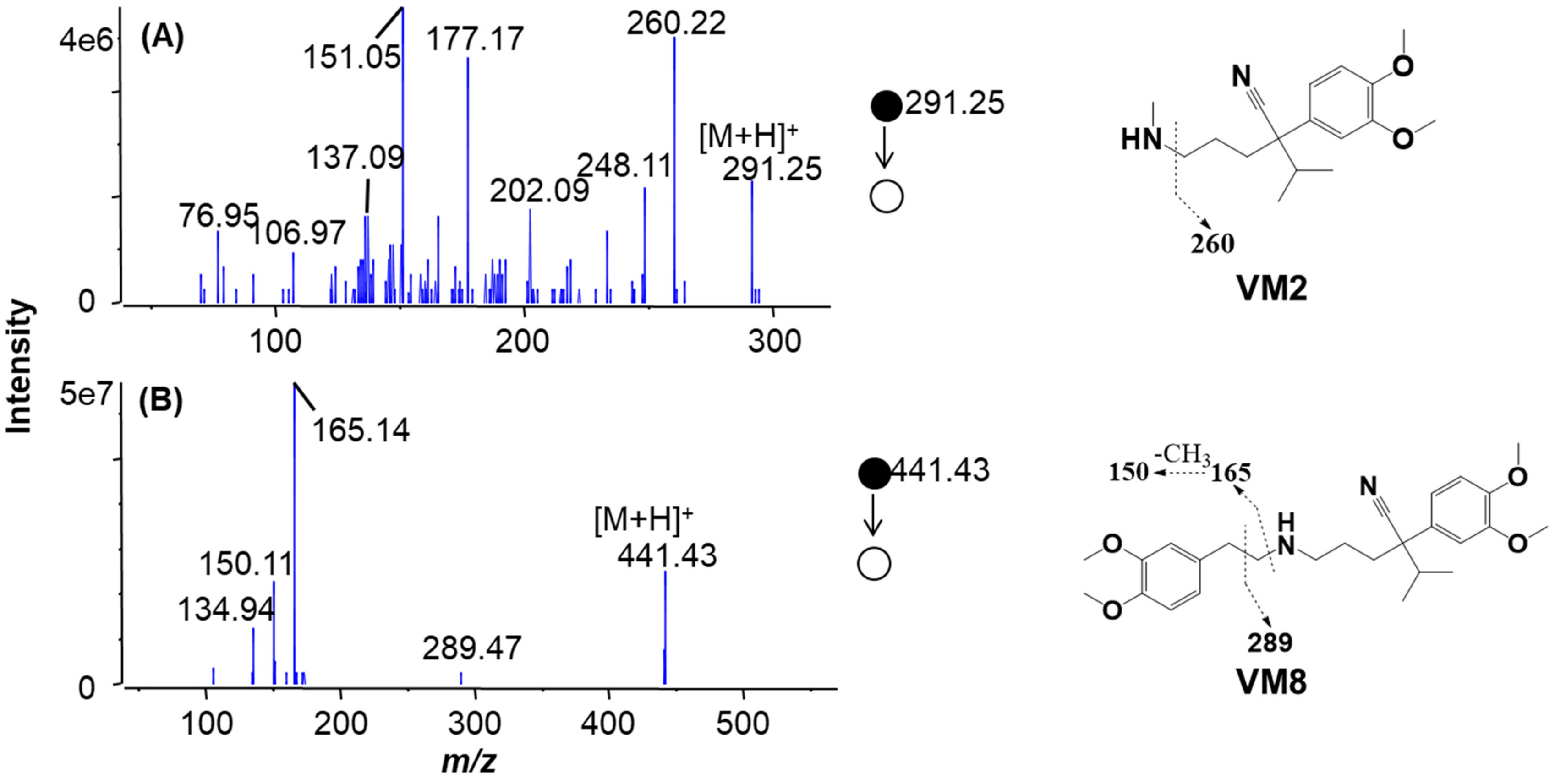

Figure S4. MS/MS spectra and fragmentations of verapamil major metabolites VM2 and VM8 acquired by LC/Qtrap. (A) VM2, and (B) VM8.

Support Table

Table S1: Summary of metabolites of verapamil detected by Qtrap and data mining of NLF and PIF

Metabolite	$[M+H]^+$ (m/z)	Identity	Rt (min)	Product ion	NLF (290 Da)	PIF (m/z 165)	NLF (m/z 260)
Parent	455	Verapamil	11.5	303, 260, 165, 150, 135, 105	√	√	√
VM1	277	N-dealkylation + N-demethylation	6.5	260, 234, 219, 202, 177, 151, 146			√
VM2	291	N-dealkylation	6.7	260, 248, 233, 202, 177, 165, 151			√
VM3	457	O-demethylation + hydroxylation	9.1	291, 260, 167, 135, 107	√		√
VM4	441	O-demethylation	9.7	289, 246, 165, 150, 135	√	√	
VM5	441	O-demethylation	10.0	303, 291, 260, 177, 165, 151, 136, 119, 91		√	√
VM6	471	Hydroxylation	10.5	453, 291, 260, 248, 181, 166, 151, 123	√		√
VM7	441	O-demethylation	10.5	303, 291, 260, 177, 165, 151, 136, 119, 91	√		√
VM8	441	N-demethylation	11.2	289, 165, 150, 135		√	
VM9	471	Hydroxylation	12.2	303,165,150,135		√	

Table S2: The MRM transitions, mass parameters and absorption wavelength (λmax) of the model compounds

	Midazolam	Dextromethorphan	Verapamil	Amodiaquine
MRM	326.2/291.1	272.2/171.0	455.3/165.0	356.3/283.1
DP (V)	120	120	90	100
CE (eV)	38	57	36	24
λmax (nm)	258	278	278	342
MIM scan ranges	50.0~450.3	50.0~450.3	150.1~550.3	50.0~450.3
